# Magnetic core-shell nanoparticles for drug delivery by nebulization

**DOI:** 10.1186/1477-3155-11-1

**Published:** 2013-01-23

**Authors:** Navin Kumar Verma, Kieran Crosbie-Staunton, Amro Satti, Shane Gallagher, Katie B Ryan, Timothy Doody, Colm McAtamney, Ronan MacLoughlin, Paul Galvin, Conor S Burke, Yuri Volkov, Yurii K Gun’ko

**Affiliations:** 1 Department of Clinical Medicine, Institute of Molecular Medicine, Trinity College Dublin, Dublin, Ireland; 2 Centre for Research on Adaptive Nanostructures and Nanodevices, Trinity College Dublin, Dublin, Ireland; 3 Department of Chemistry, Trinity College Dublin, Dublin, Ireland; 4 School of Pharmacy, University College Cork, Cork, Ireland; 5 Aerogen, Galway Business Park, Dangan, Galway, Ireland; 6 Tyndall National Institute, University College Cork, Cork, Ireland; 7 Dublin City University, Dublin, Ireland

**Keywords:** Nanomedicine, Magnetite nanoparticles, Quercetin, Drug delivery, Nebulization

## Abstract

**Background:**

Aerosolized therapeutics hold great potential for effective treatment of various diseases including lung cancer. In this context, there is an urgent need to develop novel nanocarriers suitable for drug delivery by nebulization. To address this need, we synthesized and characterized a biocompatible drug delivery vehicle following surface coating of Fe_3_O_4_ magnetic nanoparticles (MNPs) with a polymer poly(lactic-co-glycolic acid) (PLGA). The polymeric shell of these engineered nanoparticles was loaded with a potential anti-cancer drug quercetin and their suitability for targeting lung cancer cells *via* nebulization was evaluated.

**Results:**

Average particle size of the developed MNPs and PLGA-MNPs as measured by electron microscopy was 9.6 and 53.2 nm, whereas their hydrodynamic swelling as determined using dynamic light scattering was 54.3 nm and 293.4 nm respectively. Utilizing a series of standardized biological tests incorporating a cell-based automated image acquisition and analysis procedure in combination with real-time impedance sensing, we confirmed that the developed MNP-based nanocarrier system was biocompatible, as no cytotoxicity was observed when up to 100 μg/ml PLGA-MNP was applied to the cultured human lung epithelial cells. Moreover, the PLGA-MNP preparation was well-tolerated *in vivo* in mice when applied intranasally as measured by glutathione and IL-6 secretion assays after 1, 4, or 7 days post-treatment. To imitate aerosol formation for drug delivery to the lungs, we applied quercitin loaded PLGA-MNPs to the human lung carcinoma cell line A549 following a single round of nebulization. The drug-loaded PLGA-MNPs significantly reduced the number of viable A549 cells, which was comparable when applied either by nebulization or by direct pipetting.

**Conclusion:**

We have developed a magnetic core-shell nanoparticle-based nanocarrier system and evaluated the feasibility of its drug delivery capability *via* aerosol administration. This study has implications for targeted delivery of therapeutics and poorly soluble medicinal compounds *via* inhalation route.

## Background

The development of nanoparticles as controlled drug delivery and disease detection systems has emerged as one of the most promising biomedical and bioengineering applications of nanotechnology. Magnetic nanoparticles, in particular iron oxide (also called magnetite or Fe_3_O_4_) nanoparticles (MNPs) and their multifunctionalized counterparts are an important class of nanoscale materials that have attracted great interest for their potential applications in drug delivery and disease diagnosis [1-5]. Owing to the recent advances in synthesis and surface modification technologies, a variety of new potential applications have become feasible for this class of nanomaterials that may revolutionise current clinical diagnostic and therapeutic techniques.

The well-developed surface chemistry of Fe_3_O_4_ MNPs allows loading of a wide range of functionalities, such as targeting ligands, imaging and therapeutic features onto their surfaces. It is now possible to fine-tune the physical parameters of MNPs, such as size, shape, crystallinity, and magnetism [3,4]. Furthermore, MNPs have the potential for replacement or modification of the coating materials post-synthesis allowing tailoring of the nanoparticle’s surface charge, chemical groups, and overall size [4-6]. Due to their unique physicochemical properties and ability to function at the cellular and molecular level of biological systems, MNPs are being actively investigated as the next generation of targeted drug delivery vehicle. The design of such drug delivery systems requires that the carriers be capable of selectively releasing their payloads at specific sites in the body and thereby treat disease deliberately without any harmful effect on the healthy tissues. In this regard, MNPs represent a promising option for selective drug targeting as they can be concentrated and held in position by means of an external magnetic field. This allows high dose drug-loads to be delivered to a desired target tissue while minimizing the exposure of healthy tissues to the side effects from highly toxic drugs, *e.g.* chemotherapeutic agents. In addition, preclinical and clinical studies have proven them to be safe and some formulations are now FDA approved for clinical imaging and drug delivery [7]. In particular, MNPs are being extensively utilized as a magnetic resonance imaging contrast agents to detect metastatic infestation in lymph nodes (such as Combidex®, Resovist®, Endorem®, Sinerem®), give information about tumor angiogenesis, identify dangerous atherosclerosis plaques, follow stem cell therapy, and in other biomedical research [8-11]. Further, functionalized multimodal MNPs are being widely explored for numerous other biomedical applications including magnetic guidance of drugs encapsulated by magnetic particles to target tissues (for example tumor) where they are retained for a controlled treatment period [2,12-22]. Thus, fabrication of MNPs as drug conjugates has the potential to greatly benefit inflammatory disease and cancer treatments, and diagnostics.

Aerosolised therapeutics has emerged as a promising alternative to systemic drug delivery for the treatment or prevention of a variety of lung diseases such as asthma, chronic obstructive pulmonary disease, respiratory infection, and lung cancer [23-26]. An aerosol-mediated approach to lung cancer therapy holds promise as a means to improve therapeutic efficiency and minimize unwanted systemic toxicity. A number of drugs have been investigated *in vitro*, in animal models and in human trials as targeted aerosol chemotherapy for lung cancer [25-31]. A range of nebulizer systems designed for individualised and controlled preparations of therapeutic aerosols have been developed and validated (*e.g.* Aerogen’s Aeroneb® Pro nebuliser) for aerosol therapy.

**Figure 1:**
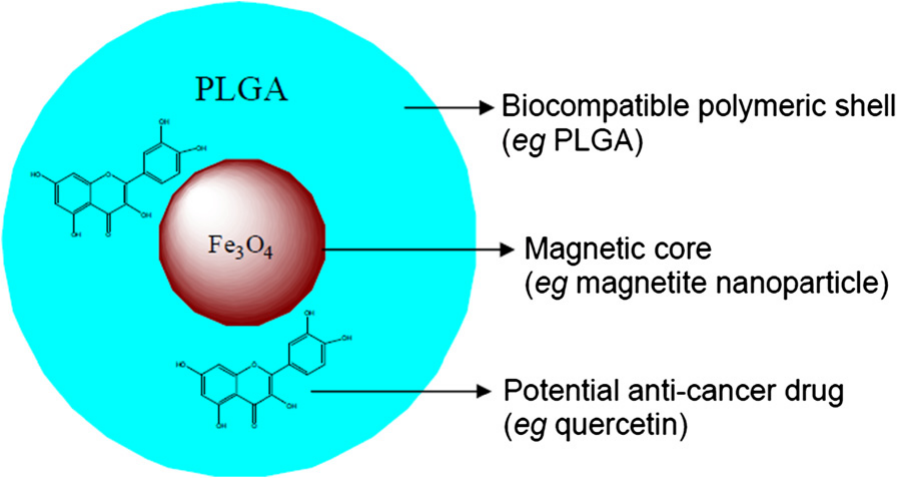
A schematic model of drug-loaded magnetic core-shell nanostructures.

The aim of this work was to establish a biocompatible MNP-based drug delivery system suitable for nebulization and inhalation targeting of therapeutics for the treatment of lung diseases. The schematic structure of the nanocarrier-drug composite is given in Figure 1. In order to improve the dispersion in aqueous medium, stability against oxidation and biocompatibility of the delivery system, MNP surface was coated with a biopolymer poly(DL-lactic-co-glycolic acid) (PLGA). In this study, we selected a poorly soluble flavonoid quercetin to act as a model drug, since it has demonstrated the potential for growth inhibition of a variety of human cancers including lung cancer [32,33]. The biocompatibility of the developed nanocarrier system was tested *in vitro* and *in vivo*, and the feasibility of a novel vibrating mesh nebulization technique was investigated for the delivery of drug-loaded MNPs to the cultured human lung cancer cells. Thus, to our knowledge, this is the first study that reports the potential of magnetic core-shell nanoparticles loaded with a poorly soluble compound quercetin for aerosol delivery by nebulization.

## Results

### Preparation and characterization of surface engineered MNPs

**Figure 2:**
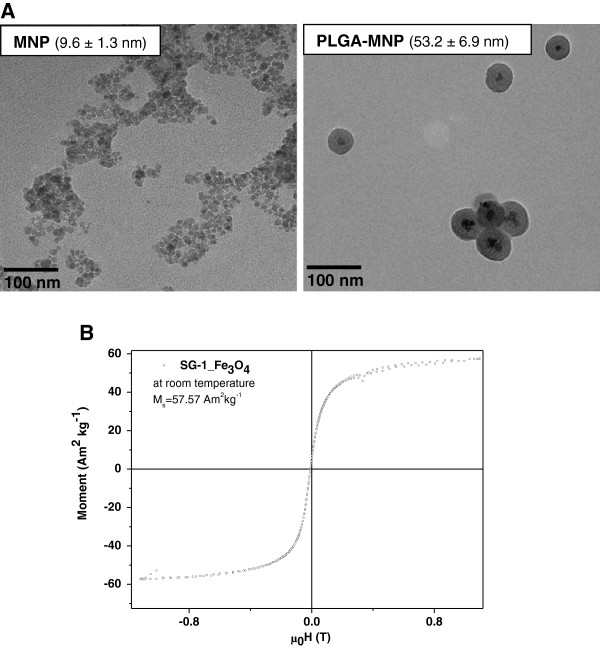
**TEM images and magnetisation curve of initial MNPs. A**. MNPs or PLGA coated MNPs (PLGA-MNP) were imaged by TEM and presented. The average size of both the MNPs was measured as indicated on the corresponding images. **B**. Magnetisation curve of initial Fe_3_O_4_ MNP at room temperature.

As evident from the analysis using transmission electron microscopy (TEM) the average size of the uncoated MNPs was 9.6 ± 1.3 nm, which was increased to 53.2 ± 6.9 nm following coating with PLGA (Figure 2A). The dynamic light scattering (DLS) measurements showed that the average hydrodynamic diameter of MNP and PLGA-MNP was 54.3 ± 8.7 nm and 293.4 ± 31.9 nm respectively. Magnetisation measurement of MNP was confirmed by its superparamagnetic properties (Figure 2B). After purification, a stock solution of 1 mg/ml was made for both the MNP preparations and stored at room temperature. The PLGA-MNP samples were stable in phosphate buffered saline (PBS) and in physiological buffers.

### *In vitro* biocompatibility analysis of engineered MNPs

**Figure 3:**
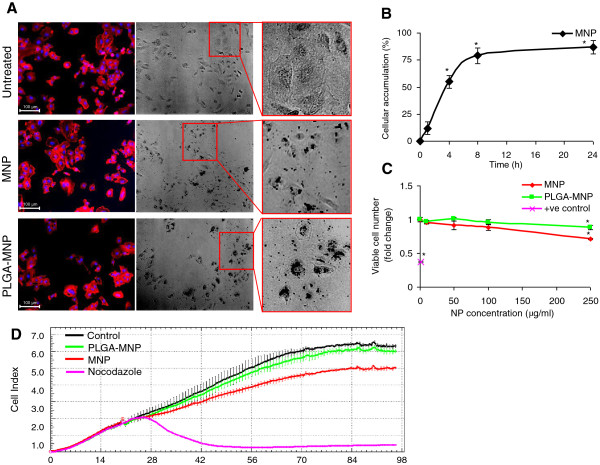
**Cellular accumulation and cytocompatibility analysis of MNPs in A549 cells. A**. A549 cells growing on 96-well tissue culture plates were incubated without or with 50 μg/ml MNP or PLGA-MNP for 24 h, washed and fixed. Adherent cells were fluorescently stained with rhodamine-phalloidin (red) to visualize cellular cytoskeleton actin and with Hoechst (blue) to visualize nuclei. Fluorescent or bright-field images acquired using an automated microscope IN Cell Analyzer-1000 are presented in the left or middle panels. Right panels show corresponding magnified images indicated by box in the middle panels. **B**. A549 cells growing on 96-well tissue culture plates were exposed to 50 μg/ml MNP for up to 24 h and imaged by IN Cell Analyzer-1000. Percentage of cells with accumulated particles were quantified by IN Cell Investigator software and presented. ^*^p < 0.05 compared to untreated control. **C**. A549 cells growing on 96-well tissue culture plates were incubated with various concentrations (ranging from 10 to 250 μg/ml) of MNP or PLGA-MNP for 24 h, washed and fixed. Cells were treated with 1 μg/ml quantum dots as a toxicity control (+ve control). HCS biocompatibility analysis was performed using IN Cell Analyzer-1000 equipped with Investigator software by quantifying cell adherence to the plates. Values in the plotted line graph are fold change in viable cell numbers ± SEM of three independent experiments in triplicate from five randomly selected fields/well containing at least 300 cells. ^*^p < 0.05 compared to untreated control. **D**. Real-time electric impedance sensing measurements of A549 cells treated with 100 μg/ml MNP, PLGA-MNP or 10 μg/ml nocodazole (as a toxicity control). Each data point is the mean Cell Index ± SEM of technical triplicates. A representative plot of three independent experiments is shown.

To investigate the biological safety of the developed nanocarriers, the cell-MNP interaction by means of cellular accumulation and their cytocompatibility on human A549 lung epithelial cells was performed *in vitro*. Initially we examined the morphology of A549 cells exposed to MNP or PLGA-MNP (50 μg/ml each) for 24 h by a cell-based automated microscope. Compared to the control untreated cells, no detectable change in the gross structure of the cytoskeletal protein actin (Figure 3A, fluorescent images) or the morphology of cells exposed to MNP or PLGA-MNP were detected (Figure 3A, bright field images). The overall shapes and sizes of cells and nuclei were within the normal variation range and there were no signs of cellular or nuclear abnormalities, membrane bound vesicles, or cell rupture (Figure 3A). No significant change in the cell morphology parameters including cell and nuclear areas and fluorescent intensities was observed following exposure to MNP or PLGA-MNP as compared to that with untreated cells. Cellular accumulation of MNP or PLGA-MNP was detected in treated cells (Figure 3A, brightfield images in the middle panel and insets in the right panel). We quantified the number of cells with accumulated MNPs over time, which included internalized MNPs and MNPs adhering to the cell surface, by In Cell Investigator software (GE Healthcare, UK). Results showed a time-dependent increase in the cellular association of MNPs, where more than 50% cells with accumulated MNPs at 4 h and over 75% cells with accumulated MNPs at 8 h and 24 h were detected (Figure 3B).

The cytocompatibility analysis of MNP and PLGA-MNP in A549 cells by high content screening (HCS) demonstrated that both the MNP preparations were non-toxic (<10% reduction in viable cell number) at concentrations up to 100 μg/ml (Figure 3C). However, a moderate but significant reduction (~25%) in the number of viable A549 cells following 24 h exposure to MNP preparations was observed at a high concentration of 250 μg/ml (Figure 3C). We further evaluated the biocompatibility of MNP and PLGA-MNP (100 μg/ml each) in A549 cells in real-time for up to 76 h using a whole cell-based electrical impedance sensing technique utilizing xCELLigance instrument (Roche Applied Science, West Sussex, UK). Untreated cells seeded onto the gold electrode array of the impedance assay E-plates (supplied by Roche Applied Science, West Sussex, UK) at a density of 5 × 10^3^ cells/well showed a continuous increase in impedance (expressed as an arbitrary unit Cell Index) over time as the cells attach, spread and form a stable confluent monolayer on the surface of the E-plate (Figure 3D). When A549 cells were treated with uncoated MNPs (100 μg/ml) at 20 h after cell seeding onto the E-plate, a low level of decrease in the Cell Index was detected over time; whereas or PLGA-MNPs did not cause any significant change in the Cell Index relative to untreated cells (Figure 3D). In contrast, a sharp decrease in the Cell Index was detected when A549 cells were treated with a toxic compound nocodazole (Figure 3D).

### *In vivo* biocompatibility analysis of engineered MNPs

**Figure 4:**
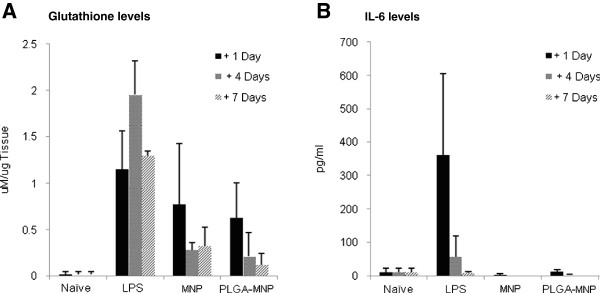
***In vivo *biocompatibility analysis of MNPs. A**. Measurement of total glutathione levels in lung tissue from mice treated with a single intranasal delivery. Lungs were harvested from treated mice at 1, 4 and 7 days post-treatment with uncoated MNP, PLGA-MNP or LPS (used as a positive control). **B**. Measurement of IL-6 levels in BAL fluid from mice treated with a single intranasal delivery. BAL fluid was obtained from treated mice at 1, 4 and 7 days post-treatment with MNP, PLGA-MNP or LPS. Data are mean ± SEM of at least 3 animals under each treatment conditions.

The biocompatibility of MNPs surface engineered with a PLGA polymer coat was also assessed *in vivo* using a mouse model. Homogenised mouse lung samples were assayed for total glutathione levels (both GSH and GSSH) as an indicator of oxidative stress after 1, 4, and 7 days post-exposure to uncoated MNP, PLGA-MNP or lipopoysaccharide (LPS, used as a positive control). Lung samples obtained 1 day after intranasal administration showed a dramatic increase in the glutathione levels in the case of all samples (Figure 4A), which may possibly be attributed to the invasive nature of intratracheal administration. However, the glutathione levels in the lung tissue were reduced 4 days after treatment with MNP or PLGA-MNP and continued to decrease thereafter (Figure 4A). In contrast, glutathione levels in mice treated with LPS remained elevated over the 7 day test period (Figure 4A). Analysis of IL-6 levels in bronchoalveolar lavage (BAL) fluid samples from treated mice measured at 1, 4 and 7 days subsequent to intranasal administration of the MNP formulations and the LPS control (Figure 4B). After 1 day, a significant increase in IL-6 levels in the case of the LPS control was observed, but this returned to background level 4 days post treatment, whereas mice treated with uncoated MNP or PLGA-MNP displayed no significant increase in IL-6 levels relative to naïve animals at any time points post-treatment.

### Efficacy analysis of quercetin-loaded MNPs delivered *in vitro* by nebulization

**Figure 5:**
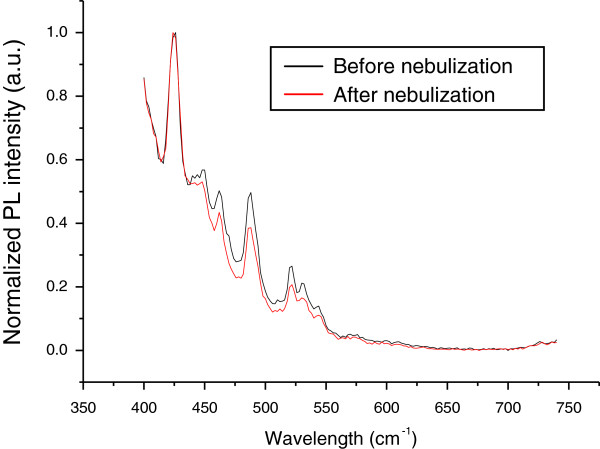
**Photoluminescence spectra of quercetin-loaded PLGA-MNP before and after nebulization.** Spectra show normalised photoluminescence (PL) intensities (a.u., arbitrary units) against wavelength (nm).

We incorporated a model drug quercetin in the PLGA-MNP and then characterized by photoluminescence before and after nebulization. No significant change in intensity and position (no shift) of the bands in the photoluminescence spectra (excited at 380 nm) was detected due to nebulization, confirming that the particles were intact and not adversely affected by the process of nebulization (Figure 5).

**Figure 6:**
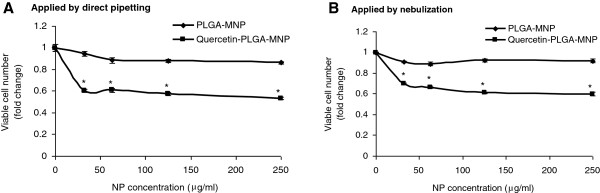
**Comparative analysis of the efficacy of quercitin-loaded PLGA-MNP delivered to A549 cells *via *direct pippetting or nebulization.** A549 cells growing on 96-well tissue culture plates were exposed to various concentrations (ranging from 10 to 250 μg/ml) of empty or quercitin-loaded PLGA-MNP by direct pippetting (**A**) or nebulization (**B**). Following treatment, cells were incubated for 24 h, washed and fixed. HCS assay was performed using IN Cell Analyzer-1000 equipped with Investigator software by quantifying cell adherence to the plates. Values in the plotted as line graph are fold change in viable cell numbers ± SEM of three independent experiments in triplicate from five randomly selected fields/well containing at least 300 cells. ^*^p < 0.05. Quercetin alone could not be used as a control due to its poor aqueous solubility.

To evaluate the therapeutic efficacy of quercetin-loaded PLGA-MNPs, they were applied to the human A549 lung carcinoma cells. A549 cells seeded in 96-well plates were exposed to varying doses of PLGA-MNP or quercetin-loaded PLGA-MNP (ranging from 31.25 μg/ml to 250 μg/ml) by direct pipetting or by nebulization and incubated for 24 h. The ability of quercetin present in the PLGA-MNP to cause cell death of A549 cells was analysed using HCS assay by quantifying the number of viable adherent cells (Figure 6). No significant change in the number of adherent cells was observed following exposure to PLGA-MNP (Figure 6). In contrast, quercetin-loaded PLGA-MNP, applied to cells either by direct pipetting (Figure 6A) or by nebulization (Figure 6B) and incubated for 24 h, significantly reduced the number of viable A549 cells. These data confirmed the *in vitro* therapeutic efficacy of the quercetin-loaded PLGA-MNP.

## Discussion

There is currently significant worldwide effort to develop, fabricate and characterize novel nanoscale materials for a variety of novel applications. In the present work, we have developed procedures to prepare novel biocompatible MNPs that possessed suitable properties for biomedical applications. Biocompatibility of the developed MNPs was characterized *in vitro* (the influence of MNPs was assessed in terms of cell viability, cellular and nuclear morphology, and observations of actin cytoskeleton) and *in vivo* (the influence of MNPs on glutathione and IL-6 secretion in mice). The developed MNPs were successfully loaded with a promising anti-cancer drug quercetin. Further, in this study we described a novel method of drug-loaded nanoparticle delivery to lung cancer using aerosols. The optimised proof-of-concept nanoplatform documented in the present study can further be exploited to load functionalities onto the MNP surfaces *via* various mechanisms with broad implications for pharmacotherapies, drug delivery and molecular imaging.

A targeted drug delivery system requires the design of carriers capable of selectively releasing their payloads at specific sites in the body. Although a number of nanosize materials are being exploited for drug delivery purposes including for example PLGA nanoparticles [34,35], MNPs represent a highly promising option for selective drug targeting as they exhibit a wide variety of desirable attributes. In particular, they can be concentrated and held in position with the aid of an external magnetic field. The deposition, accumulation, and retention of drug-conjugated MNPs in target tissue can thus be enhanced by magnetic guidance. Such magnetic targeting allows very concentrated drug doses to be delivered to specific area while minimizing the exposure of healthy tissues to uncontrollable highly toxic therapeutic substances; *e.g.* chemotherapeutic agents. Moreover, the superparamagnetic behaviour of MNPs provides multifunctional effects such as controlled heating capability under an alternating magnetic field, which has demonstrated tremendous promise as theranostics for the detection and treatment of cancer [7,9,36,37]. In addition, iron oxides occur naturally in human heart, spleen and liver [38], which supports the biocompatibility and non-toxicity of MNPs at a physiological concentration. Due to the above-mentioned favorable features and versatility, in our opinion, Fe_2_O_3_ MNPs would serve as an excellent core material for a nano carrier system particularly suitable for the controlled aerosol drug delivery.

To date a wide variety of MNPs have been developed by several researchers, differing in size and type of coating materials used [7-13,39-42]. Some preparations are currently in preclinical or clinical use in intracellular hyperthermia treatments and MRI contrast agents [7,9]. It is important to note that in order to improve the size distribution of MNPs and prevent their aggregation in aqueous solution these nanoparticles have to be coated with materials that keep particles apart. However, there is quite contradictory information on the effect of magnetic nanoparticles - biopolymer core-shell structures on cytotoxicity. It has been suggested that upon internalization, the coating shell on the MNPs may be broken down yielding particle chains and aggregates, which may influence biological processes [42,43]. In this study, we modified the surface of Fe_3_O_4_ MNPs by coating them with a biocompatible polymeric material PLGA, which has been proven to be beneficial for nanoparticle coating purposes with no measurable toxicity reported [42,44].

In order to evaluate the biocompatibility of developed MNPs, we performed a series of *in vitro* assays using a human lung alveolar epithelial cell line A549 and *in vivo* studies using normal Balb/c mice. The carcinoma-derived A549 cells are a well-characterised *in vitro* lung epithelial model and have been extensively used for assessing cytotoxicity, including nanomaterials-induced cytotoxicity [45-47]. Additionally, A549 cells display similar uptake and toxicity of nanoparticles as compared to normal primary lung epithelial cells, although both cell types respond differentially for the release of cytokines involved in inflammatory reactions [48]. Based on these reports and our data from *in vitro* as well as *in vivo* experiments presented here, we expect that the developed MNPs will have similar effect(s) on normal lung epithelial cells in terms of their cytocompatibility. However, a detailed characterization of MNPs on normal lung cells should be performed before their potential clinical applications in drug delivery.

We employed the use of HCS in combination with an impedance-based assay for the biocompatibility analysis of MNP preparations. The HCS assay utilizes a novel quantitative imaging technique and offers rapid analysis of toxicity (if any) at cellular level [46,47]; whereas, impedance sensing allows a kinetic profile of cytotoxicity (if any), and maps the processes that cells undergo when challenged with nanoparticles such as MNPs [49,50]. Since the insulating properties of cells are based on whole cell structure, cellular responses such as cell death, proliferation, spreading and attachment can be detected by impedance measurements [49,50]. This cell-based label-free non-invasive detection method thus not only provides toxicity data, but also can identify a time-frame during which further targeted analysis can be performed. Both HCS and impedance measurement assays confirmed that MNPs developed in the present work were not toxic to A549 cells up to a concentration of 100 μg/ml, although a high concentration of 250 μg/ml were moderately toxic.

As described, we selected quercetin as a model drug. Quercetin is one of the most prevalent as well as thoroughly studied dietary flavonoids with several biological and pharmacological properties. Evidence indicates that quercetin has a variety of anti-cancer mechanisms, including anti-proliferative, pro-apoptotic, cell signalling effects, and growth factor suppression, as well as potential synergism with some chemotherapeutic agents [32,51]. Quercetin also exhibits anti-inflammatory, anti-oxidant, and anti-viral activities [33]. Moreover, it has a role in reversing drug resistance, re-sensitizing cancer cells to some chemotherapeutic agents and in potentiating the effectiveness of some chemotherapeutic agents [52]. However, realizing the therapeutic benefits of quercetin in the clinical setting is hampered by its low solubility (~ 2%) in aqueous medium and poor absorption in the body. Thus, the low bioavailability and poor solubility in aqueous medium are major concerns associated with the therapeutic application of quercetin [51,52]. Similar limitations apply to experimental evaluation of quercetin’s effect on cultured human cells in biological medium, and therefore quercetin alone could not be used for comparison in the present study. The MNP carrier system developed in the present study was appropriate in this regard; and therefore, the ability of quercetin to inhibit lung cancer cell growth was evaluated in comparative analysis of non-functionalized and drug-loaded PLGA-MNPs.

Administration of the MNPs resulted in elevated levels of GSH in lung tissue, an indicator of oxidative stress [53], but this was not observed to be consistently elevated over the follow-up period of 7 days unlike the LPS control. IL-6, which acts as both a pro-inflammatory and anti-inflammatory cytokine [54], and is secreted by T-cells and macrophages to stimulate an immune response during infection and after tissue trauma was also investigated as a marker of immune response. The LPS caused a significant increase in IL-6 levels in BAL samples 1 day after treatment as expected, but this was not replicated in the case of the MNPs. In fact the IL-6 levels in BAL samples from mice exposed to MNP or PLGA-MNP were comparable to those observed after administration of normal saline solution in control groups. IL-6 levels in blood plasma (data not shown) were of a much lower level and more variable indicating the localized nature of the response in the pulmonary tissue. This is in agreement with the *in vitro* results discussed above. Previously it has been shown that intranasal delivery of iron nanoparticles can lead to an increase in inflammatory markers including IL-6 [55]. PLGA particles themselves have been shown to have a low propensity to cause immune responses when delivered directly to the lung [56]. The biocompatible MNPs developed in this work may also be potentially exploited for targeting using external magnetic fields as demonstrated recently in nebulized mice [57].

Regional chemotherapy has been proposed as a treatment modality in a number of disease situations in order to increase exposure of the target tissues to the drug, while minimising systemic side-effects. Administration of drugs directly *via* inhalation allows localized drug delivery to the lungs and airways with smaller doses and minimal systemic toxicity [58]. An additional reason for maximising total deposition and targeting drugs to their desired location is to improve the cost effectiveness of drug delivery [59]. There is now increasing evidence to support the role of inhalation therapeutics in the treatment of various lung diseases. For example in lung cancer, nebulization therapeutics could be useful in 1) unresectable bronchioloalveolar carcinoma or main bronchus carcinoma with limited invasion, 2) endobronchial tumour relapse after surgery, 3) *in situ* carcinoma or synchronous, or 4) metachronous lesions in patients where a lesion has already been detected. However, few studies have documented the feasibility of applying nanotechnology for inhalation delivery of anticancer agents [60]. Therefore, new aerosol delivery technologies are currently being developed to meet these goals of improved targeting, reduced waste and improved patient compliance. Vibrating mesh-based nebulizers (*e.g.*, Aerogen nebulizer) can allow for sensitive tracking of flows or pressures during breathing manoeuvres and offer the potential for high efficiency delivery of aerosolized medications. These nebulizers have been used for breath actuated high efficiency aerosol delivery during mechanical ventilation of humans and rodents [61,62]. Appropriate aerosol actuation during defined portions of the breath, allow for aerosol-free intervals, if required, thus avoiding drug deposition in the dead space of the patient interface, and even targeting of specific potions of the lung, *e.g.,* introducing the aerosol in a small bolus at the end of inspiration to target the upper airways. Although the customizable vibrating mesh-type nebulizers have not been applied clinically to deliver MNP-based cancer therapeutics to the lung heretofore, the present study provides proof of principle for such targeting.

## Conclusion

Here, we report the development of a surface engineered magnetic core-shell nanoparticle-based drug delivery system designed for aerosol therapy of lung diseases. We present a series of *in vitro* and *in vivo* investigations that were carried out to evaluate the biocompatibility of the developed nanocarrier and the feasibility of pulmonary delivering quercetin-loaded MNPs by nebulization. The data presented here demonstrate inhibition of lung adenocarcinoma growth by aerosol delivery of quercetin loaded in the PLGA-MNPs. Further *in vivo* studies are required to determine the optimal dosage and frequency of aerosol administrations and to assess the anticancer effect of nanoencapsulated aerosolised chemotherapy on established tumours. With the on-going efforts to enhance MNP’s targeting ability, endow more functions and administration routes, their future holds great promise for advanced drug delivery applications.

## Methods

### MNP preparation

FeCl_2_ (12 mM) and FeCl_3_ (24 mM) were dissolved in 25 ml HCl (0.4 M) solution. The precursors were added drop-wise to a degassed 0.5 M NaOH solution at 40°C. The mixture was stirred for 1 h, and then cooled to room temperature. The MNPs were subjected to magnetic separation and then washed repeatedly with water until neutral pH was reached. Next, MNPs were surface coated by o/w emulsification of 4 ml acetone:dichloromethane (1:2) contained 100 mg PLGA (Sigma-Aldrich Ireland Ltd., Wicklow, Ireland), 50 μl of 10 mg/ml MNP, and 20 mg quercetin (where appropriate). The mixture was added to 12 ml of 0.3% polyvinyl alcohol aqueous solution (15,000 g/M). The mixture was emulsified under a sonic tip for 30 seconds, the emulsion was then added to 50 ml of 0.3% polyvinyl alcohol solution and stirred overnight to remove the organic phase. The suspension was then centrifuged at 3000 *g* and subsequently re-dispersed in water several times to remove excess polyvinyl alcohol.

### Transmission electron microscopy (TEM)

Samples for TEM were prepared by deposition and drying of a drop of the powder dispersed in ultrapure water onto a formvar coated 400 mesh copper grids. High resolution TEM images were acquired using an FEI-Titan TEM.

### Dynamic light scattering (DLS) measurements

DLS measurements were performed using a Malvern Zetasizer Nano Series V5.10. The concentration of samples used for these measurements typically corresponded to an approximate absorbance of 0.2 nm. Three measurements were usually taken for each sample and then averaged.

### Cell culture and treatments

Human alveolar epithelial cells (A549 cell line, European Collection of Cell Cultures, Salisbury, UK) were cultured as described [47]. Briefly, cells were cultured in Gibco® Ham’s F12 medium supplemented with 10% (v/v) foetal bovine serum, 10,000U penicillin and 10 mg/ml streptomycin in 5% CO_2_ at 37°C in a humidified incubator. For experimentation, cells were seeded in 96-well plates at the density of 4 × 10^3^ cell/well and allowed to grow overnight prior to treatment. Nanoparticles were dispersed in PBS to make a stock solution of 1 mg/ml and then diluted in cell culture medium prior to administration to the cells. Serial dilutions were established by mixing equal volumes of particle suspension and cell culture medium followed by vigorous vortexing, and applied to the cells immediately. The cell culture media and supplements were from Life Technologies Corporation (Bio-Sciences, Dublin, Ireland).

### High Content Screening (HCS) and analysis

HCS protocols for nanotoxicity studies have been optimized and established in our laboratory as described [46,47,63-68]. Briefly, A549 cells were seeded in 96-well plates (4 × 10^3^ cells/well), exposed to various concentrations of MNP preparations for varying time-points (as indicated in the text and corresponding figure legends) at 37°C and 5% CO_2_. After washing three times with PBS, cells were fixed by incubating them for 20 min with 3% paraformaldehyde. Adherent cells were then fluorescently stained with rhodamine labelled phalloidin to visualize the cellular morphology and Hoechst to visualize the nuclei. Plates were scanned (five randomly selected fields/well) using an automated microscope IN Cell Analyzer 1000 (GE Healthcare, UK) and the acquired images were automatically analysed by IN Cell Investigator (version 1.6) software using multi-parameter cytotoxicity bio-application module (GE Healthcare, UK).

### Real-time impedance sensing

The dynamic monitoring of electrical impedance (which depends on cell number, degree of adhesion, spreading and proliferation of the cells) to determine cytotoxic effects of MNPs was performed using Real-Time Cell Analyzer DP instrument as per manufacturer’s instructions (xCELLigance system, Roche Applied Science, West Sussex, UK) and described previously [47,68,69]. Briefly, A549 cells were seeded at a density of 5 × 10^3^ cells/well in 100 μl medium in the E-Plates 16 (cross interdigitated micro-electrodes integrated on the bottom of 16-well tissue culture plates by micro-electronic sensor technology) and allowed to attach onto the electrode surface over time. The electrical impedance was recorded every 15 minutes. At 20 h time point, when cells adhered to the well properly, they were treated with MNP preparations in triplicate and monitored for a further 76 h to record changes in cell behaviour. To ensure the MNP preparations did not interfere with the impedance measurements, control wells containing medium only and corresponding MNP samples were run in parallel. The cell impedance, expressed as an arbitrary unit called the ‘Cell Index’, were automatically calculated on the xCELLigence system and converted into growth curves.

### MNP delivery to lung epithelial cells by nebulization

The delivery of the engineered MNP preparations to A549 lung cancer cells by nebulization was performed using a proprietary vibrating mesh-type nebulizer (Aeroneb® Pro nebulizer system, Aerogen, Galway Business Park, Ireland) (volumetric mean diameter 3.65 μm and nebulizer flow rate 0.190 ml/min with normal saline) as per manufacturer’s instructions. Briefly, A549 cells were seeded in 96-well plates (4 × 10^3^ cells/well) and allowed to adhere for 24 h. Cells were exposed to nanoparticles either by directly pip petting or nebulizing media containing varying concentrations of MNPs (as indicated in the text and corresponding figure legends) into the wells. Following exposure, cells were incubated for a further 24 h and the number of viable adherent cells was quantified by HCS assay as described above.

### *In vivo* biocompatibility testing of MNPs

Six to eight week old female Balb/c mice (Harlan, UK) were allowed to acclimatise for two weeks before the initiation of the study. For all the *in vivo* experiments, ethical approval was obtained from the internal Ethics Committee of University College Cork, Ireland. Mice were treated intranasally with 50 μl solution containing 1 mg/ml of MNP preparations. LPS (50 μg/ml) was administered similarly as a positive control, since it is known to be a potent initiator of acute lung injury [70]. Mice were euthanized at 1, 4 and 7 days post-treatment and the lungs were excised. Tissue was homogenised in 50 mM MES buffer followed by centrifugation. The supernatant was then deproteinated and glutathione levels were determined using a Glutathione Assay Kit as per manufacturer’s protocol (Cayman Chemical Company, MI, USA). BAL fluid was obtained to assess the IL-6 levels. Mice euthanized at 1, 4 and 7 days post-treatment were dissected to expose the trachea. A small incision was made in the trachea and 1 ml of cold sterile saline was loaded into the lung and immediately removed. This was centrifuged at 3000 *g* for 10 min to remove cellular material. The supernatant was assayed for IL-6 levels using an IL-6 ELISA kit as per manufacturer’s protocol (eBioscience, UK).

### Statistical analysis

Each experiment was repeated a minimum of three times. The data are expressed as mean ± SEM. For comparison of two groups, *p*-values were calculated using the two-tailed student’s *t*-test. In all cases, statistical significance was accepted at a level of *p*-values < 0.05.

## Abbreviations

BAL: Bronchoalveolar lavage; DLS: Dynamic light scattering; HCS: High content screening; LPS: Lipopoysaccharide; MNP: Magnetic nanoparticles; PBS: Phosphate-buffered saline; PLGA: Poly(lactic-co-glycolic acid); TEM: Transmission electron microscopy.

## Competing interests

The authors declare that they have no competing interests.

## Authors’ contributions

NKV carried out *in vitro* cytocompatibility studies, participated in the conception of the study, and drafted the manuscript. KCS performed *in vitro* nebulization experiments. AS and SG designed and synthesized all the nanoparticles used in this study. KBR oversaw the *in vivo* experimentation and both KBR and TD were involved in the conception and performance of the *in vivo* studies. CM, RM, PG and CSB participated in the critical assessment of the data, and helped to draft the manuscript. YV and YKG supervised the study, participated in the data analysis and drafting of the manuscript. All authors have read and approved the final manuscript.
